# Supplementary material for: Protocol for evaluation of Movember’s scaling what works grant funding program: Supporting the delivery of mental health interventions for men & boys in Australia, Canada and the United Kingdom
Source: PLoS One. 2026 Jan 7;21(1):e0339006. doi: 10.1371/journal.pone.0339006 (PMC12779127; doi:10.1371/journal.pone.0339006)
Supplement: S1 File — (DOCX) [file pone.0339006.s001.docx]

S1 File. Evaluation map: Evaluation domains, questions and data sources

| Evaluation domains and questions | | Project delivery & administrative data | Costing survey | Participant surveys | Implementation focus group | Scalability Assessment | Project & evaluation teams’ engagement |
| --- | --- | --- | --- | --- | --- | --- | --- |
| **DOMAIN 1- Implementation: To what extent have projects been implemented as intended?** | | | | | | | |
| **Reach, accessibility, and adoption** | Did the individual projects reach their target population and provide access to their intervention, as planned?   - 1. In terms of the number of individuals who accessed information on the intervention, signed up to the intervention, participated in and completed the intervention   2. According to the characteristics of the individuals | 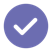 |  |  |  |  |  |
|  | Were the individual projects able to deliver their intervention with delivery sites, as planned?   - 1. In terms of the number of delivery sites that engage with the project, the number of delivery sites that delivered the intervention and the number of delivery sites in line with planned activities   2. According to the characteristics of the delivery sites | 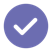 |  |  |  |  |  |
|  | How accessible did the participants find the intervention they received? |  |  | 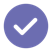 |  |  |  |
|  | To what extent did the participants engage with the intervention? | 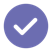 |  | 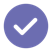 |  |  |  |
| **Implementation delivery** | How was a gendered lens embedded in the project and across project components? |  |  | 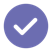 | 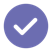 | 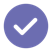 | 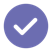 |
|  | To what extent were the interventions adapted during the delivery of the projects? |  |  |  | 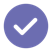 |  | 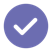 |
|  | To what extent were the interventions delivered as intended (with fidelity) as defined by the project teams?   - 1. To what extent did the project monitor fidelity as planned? | 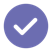 |  |  | 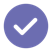 |  |  |
|  | To what extent were the interventions delivered considered acceptable by the workforce delivering the intervention?   - 1. Did this vary across delivery sites?   2. What were the main factors associated with acceptability?   3. Did the delivery workforce receive enough support to deliver the intervention? |  |  |  | 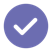 |  | 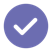 |
|  | To what extent were the interventions delivered considered acceptable by participants? |  |  | 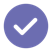 |  |  | 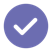 |
|  | Which implementation strategies did the projects plan to use? |  |  |  | 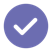 |  | 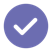 |
|  | Which implementation strategies did the projects use? How did they compare with the planned implementation strategies? |  |  |  | 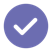 |  | 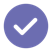 |
|  | Which barriers and enablers to implementation were identified? |  |  | 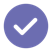 | 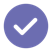 |  | 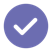 |
| **DOMAIN 2- Effectiveness: To what extent have participant outcomes changed over time – within projects & across Scaling What Works?** | | | | | | | |
|  | To what extent was the SWW program associated with changes in participant wellbeing? |  |  | 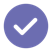 |  |  |  |
|  | To what extent were the projects associated with changes in the following medium to long term outcomes?   - 1. Participant wellbeing (all projects)   2. Other key outcome identified by the project team (max of two outcomes per project) |  |  | 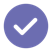 |  |  |  |
| **DOMAIN 3- Cost-effectiveness: Do the calculated cost of implementation outweigh the benefits – within projects & across Scaling What Works?** | | | | | | | |
| **Resources used and delivered** | How much time was spent by the delivery workforce to deliver the intervention?   - 1. How did the time spent by the delivery workforce differ from what was expected? |  | 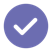 |  |  |  | 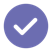 |
|  | Which training and other operational activities were delivered as part of the intervention?   - 1. How did this differ from the expected delivery of operational activities? |  | 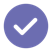 |  |  |  | 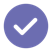 |
|  | Which infrastructure was delivered as part of the project?   - 1. How was this different than the infrastructure expected to be delivered? |  | 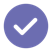 |  |  |  | 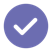 |
| **Cost analysis** | How much did the scale up of the interventions cost to deliver overall for each project?   - 1. Per delivery site?   2. Per participant? | 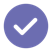 | 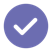 |  |  |  |  |
|  | To what extent did the costs of delivering the scale up of the intervention differ from the planned costs?   - 1. For different type of activities   2. For different cost types   3. Which type of activity or cost types varied the most? |  | 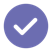 |  |  |  | 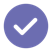 |
| **Cost effectiveness** | How cost-effective was the intervention delivered by the projects?   - 1. With regards to increasing the wellbeing of participants   2. How does the monetary value attached to the change in wellbeing compared to the cost of scaling up the intervention? | 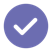 | 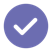 | 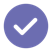 |  |  |  |
|  | How does the estimated cost-effectiveness of the intervention delivered compare to the expected cost-effectiveness? |  |  |  |  |  | 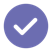 |
| **DOMAIN 4- Sustainability & scalability: To what extent are projects sustainable & ready to scale further?** | | | | | | | |
| **Sustainability** | To what extent did the intervention appear integrated within the delivery setting? |  |  |  | 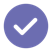 | 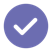 | 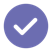 |
|  | Were any consequences linked to the intervention identified as impacting the delivery setting associated with the intervention (e.g., increase demand for other services, safeguarding resourcing, vicarious trauma, increased workload)?   - 1. Did these consequences appear to increase risks within the delivery context?   2. Did these consequences appear to increase resourcing needs in the delivery context?   3. Did the delivering setting appear to have access to enough funding to manage these consequences? |  |  |  |  | 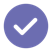 | 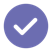 |
| **Scale up success** | To what extent did the project deliver the scaling of their interventions as planned (considering the implementation, effectiveness, and economic evaluation findings)? | 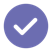 | 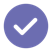 | 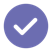 | 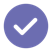 | 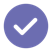 | 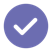 |
|  | To what extent did the project develop awareness and understanding of these political, strategic and structural contextual factors impacting the scalability of their intervention? |  |  |  |  | 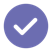 | 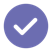 |
| **Scalability factors** | Which scaling implementation strategies did the project plan to use? |  |  |  | 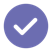 | 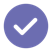 |  |
|  | Which scaling implementation strategies did the project use? How did this compare with the scaling implementation strategies planned? |  |  |  | 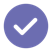 | 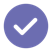 | 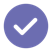 |
|  | Which barriers and enablers to successfully scaling were identified? |  |  |  | 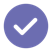 | 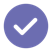 | 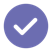 |
|  | What were the determinants of scalability identified across the SWW program? |  |  |  | 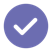 | 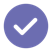 | 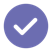 |
| **Scalability assessment** | How could the project team improve the scalability of their intervention? |  |  |  | 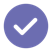 | 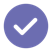 | 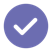 |
|  | How scalable do the interventions of each of the project appear? | 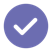 | 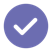 | 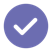 | 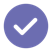 | 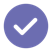 | 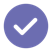 |
