# Supplementary material for: Protocol for evaluation of Movember’s scaling what works grant funding program: Supporting the delivery of mental health interventions for men & boys in Australia, Canada and the United Kingdom
Source: PLoS One. 2026 Jan 7;21(1):e0339006. doi: 10.1371/journal.pone.0339006 (PMC12779127; doi:10.1371/journal.pone.0339006)
Supplement: S2 File — (DOCX) [file pone.0339006.s002.docx]

S2 File. Brief description of the 17 projects funded by the SWW program

| Project name | Country | Target group | Delivery setting | Brief intervention description | SWW delivery and scaling focus |
| --- | --- | --- | --- | --- | --- |
| Dads Tuning into Kids | Australia | Fathers of 3- to 12-year-olds who are already participating in fathering groups. | Community | A 7-session, manualised, group-based parenting program aiming to help fathers explore their role in children’s development. Through emotion coaching, the program supports fathers’ emotional awareness and regulation, improves wellbeing and supports building children’s emotional competence through play. | To deliver the program to fathers in hard-to-reach areas (e.g. socially/ geographically isolated or incarcerated). |
| South-Western & Western Sydney Men's Mental Health & Gambling Harm Prevention Project | Australia | Men aged 16 and above from Western and South-Western Sydney who are experiencing (or at-risk of experiencing) gambling harm and associated mental health challenges. | Community | Reclink Australia delivers flexible sport and recreation program across the country. This program is specifically targeted at men experiencing or at risk of gambling harm or addiction. Working with delivery partners, the program comprises flexible, recreational sport sessions which participants can join on a drop-in basis, with structured 'doorstop’ activities focusing on mental wellbeing, social cohesion and gambling/ gambling-related harm. | South-Western and Western Sydney is a region with high proportion of new/recent migrants, who are at greater risk of gambling-related harm. |
| Western Bulldogs Sons of the West Men's Health Program: Engaging with CALD communities | Australia | Culturally and linguistically diverse (CALD) men (e.g., South Asian, Vietnamese, and multiple groups within the African-Australian diaspora) of all ages living in the West of Melbourne. | Community | Sons of the West is a 10-week place-based health program which leverages the brand of the Western Bulldogs Football Club to foster engagement of men from all walks of life. Delivered in conjunction with local councils and community organisations, the program aims to improve wellbeing, increase physical activity, social connection and decrease psychological distress. Participants in SOTW are also more able to undertake health practices and are more willing to seek healthcare support. | Program delivery under the SWW initiative focuses specifically on engaging culturally and linguistically diverse communities in the western region of Melbourne. |
| Top Blokes Mentoring Program | Australia | Male students aged 10 to 17 residing on the Sunshine Coast who are at risk of, or currently experiencing, mental health challenges, social disconnection, or have a diagnosed emotional or behavioural condition. | Schools | Top Blokes mentoring program comprises weekly, school-based workshops over a school semester for young males to develop the tools to build their own mental wellbeing and to support their friends in need. The mentoring program provides a supportive learning environment to cover key topics and issues that develop positive habits support mental resilience and increase knowledge about sexualities, discrimination, stereotyping and awareness of support services. | Delivery under SWW focuses on the delivery of the program to more schools by expanding to the Sunshine Coast region in Queensland. |
| Our Futures | Australia | Young men aged 13 to 17 in participating schools and youth services across Australia. | Schools | Our Futures is a suite of prevention programs delivered within schools which informs secondary students about substance use and mental health. The program improves participants’ awareness of physical and mental health through engaging with peer-led messages about drug and alcohol use and/or mental health. The program utilises interactive cartoon storyboards to empower proactive problem-solving and develop emotional management skills to help make decision-making related to alcohol and drug use, health, and wellbeing. | Delivery under SWW focuses on the delivery of the program to more schools in New South Wales and potentially other states in Australia. |
| Preventure | Australia | Young men aged 13 to 15 in participating schools and youth services across Australia who are identified as high-risk (high scores on measures of at least one of four personality traits associated with an increased risk of substance use and mental health problems). | Schools | Preventure is a personality-targeted prevention program designed to upskill adolescents to cope with emotions and risk-taking behaviours. It comprises two 90-minute workshops which focus on motivating adolescents to understand how their personality leads to certain emotional and behavioural reactions and identify adaptive ways of coping. The program aims to empower adolescents to make decisions that promote their wellbeing and reduce their risk of substance use and mental health problems, including suicidal ideation. | Delivery under SWW focuses on the delivery of the program to more schools in New South Wales and potentially other states in Australia. |
| Edge of the Present | Australia | Young men under 25 who are socially disadvantaged and/or living in regional and remote communities who face a wait for services. | Community | Edge of the Present is a single-session, immersive Virtual Reality suicide prevention program supporting young male participants to develop positive habits and build mental resilience. The program targets positive image generation as a key ‘mechanism of therapeutic change’, engaging directly with feelings and sensation rather than cognition. While the nominated dosage is a minimum of one (single) session, the program team has stated an ideal dosage as one weekly session over the span of four weeks. | SWW delivery involves the engagement and recruitment of additional sites to deliver the program within. The scaling effort will also entail a combination of technical redevelopment to establish the program’s potential as a suicide prevention tool in accessible everyday settings. |
| Scaling WiseGuyz to Youth Criminal Justice Settings | Canada | Young men aged 12 to 17 years who are in conflict with the law and are involved at John Howard Society sites, or other partner sites, in Canada. | Youth justice | WiseGuyz is an upstream health promotion program that supports young male participants to explore health-harming gender norms and examine their gender identity as it relates to the influence of stereotypical male role norms. Sessions run weekly across four modules, with a minimum effective dosage of 7 attended sessions to effect change in the internalisation of masculine norms and awareness of dating abuse. | The delivery of WiseGuyz under the SWW program focuses on expanding to youth justice sites in Alberta and Ontario. |
| RISE YBMen Toronto | Canada | African, Caribbean, and Black young men aged 16 to 30 who have experienced the homicide of a family member or friend. | Community | RISE YB Men Toronto is an 8-week, social media-based program delivering mental health education tailored to the issues and experiences of African, Caribbean, and Black men who have experienced the homicide of family or friends. The program aims to improve participants’ short-and long-term mental health outcomes (e.g. reducing depressive symptoms) and develop sustainable support networks that help individuals cope with their grief. | The program is adapted from the original YBMen project delivered in the United States and Australia, to focus on trauma-related content for young men who have experienced the homicide of a friend or family member. |
| Collective Resilience by Working with Men in Sports and Community Settings | United Kingdom | Men living in the UK, with priority given to individuals from socially disadvantaged backgrounds. | Community | Collective Resilience is a workshop-based program aimed at encouraging social connection among men and encouraging them to start new conversations and rethink key topics around masculinity, culture, and mental health issues. There are three workshop styles in which Collective Resilience can be delivered: 1. Workshop program: Three two-hour sessions (team talk, collective resilience and mental wellbeing allyship)  2. Professional development in facilitation of the program in potential agents of change (e.g., youth workers, community leaders): Seven workshop components plus supervision  3.Inclusive and supportive leadership training workshop: One five-hour session for 30 leaders selected from participant groups | Collective Resilience has previously been delivered in community groups and sporting contexts. SWW aims to expand delivery to socially disadvantaged groups who are less likely to have the opportunity to discuss gender norms and how these norms impact them and others. |
| They Call Me Dad | United Kingdom | GBTQ+ parents and prospective parents who are experiencing challenges associated with the transition to parenthood. | Community | They Call Me Dad is an early intervention support program aimed at improving the mental health and wellbeing of GBTQ+ parents and intended parents. This is achieved in part via a dedicated app ‘Baby Buddy’ which provides resources and mental health supports (e.g., crisis text line, face-to-face sessions) to address specific needs during the adoption, surrogacy, and parenting journey. | Delivery under the SWW program focuses on an extensive co-production and development phase of GBTQ+ specific content for the Baby Buddy app, which will then be rolled out to the wider GBTQ+ community. |
| Achieving Active Lives | United Kingdom | Men experiencing early issues with their mental health, with a particular focus on communities experiencing social disadvantage and unemployment. | Community | The Achieving Active Lives (AAL) program encourages and engages men to learn and apply behavioural change techniques, increasing self-esteem and confidence, utilising physical activities to improve their mental, physical, social, and emotional health and wellbeing. The program involves a four-week readiness to change preparation (including building skills in self-management, self-esteem, engagement, and behaviour change), followed by weekly, then fortnightly, one-to-one in-person hourly meetings with a behaviour change practitioner over 16 weeks. Participants also receive access to community-based exercise activities, peer support groups and training. | The SWW delivery model for AAL involves expansion to two large cities in Scotland (Dundee and Glasgow) that neighbour regions where the program was originally implemented. |
| Sport in Mind | United Kingdom | Men aged 16 and above who are struggling with their mental health and are not engaging in traditional mental health services. | Community | Sport in Mind is a bespoke, insight driven initiative based on 12 years of delivery in partnership with the National Health Service (NHS). The program involves weekly accessible and supportive sports sessions (offered on a drop-in basis and participants can attend for as long as desired) focusing on combating isolation, early mental health issues and suicide prevention. It aims to improve participants’ mental and physical health. Training and volunteering opportunities will also be provided. | Program delivery under SWW will involve targeting men from socially disadvantaged communities and is focused on scaling up from their Thames Valley mental health sports interventions model to reach all counties in Southern England. |
| Growing2gether | United Kingdom | At-risk young men aged 13 to 16 years in Scottish Highland areas, Dundee/Aberdeen who are in remote and/or deprived areas. | Schools | Growing2gether is a 16–18-week preventative intervention program which pairs disadvantaged young people as mentors to nursery children needing additional support. The program combines this experience with classroom coaching in interpersonal skills and improves mental health and wellbeing outcomes (e.g. anxiety and depression symptoms). | Building on existing program implementation, the focus of SWW delivery is on expanding the program to more isolated and socially disadvantaged communities. |
| Becoming a Man | United Kingdom | Young men aged 12 to 16 years from six schools based in London boroughs Lambeth and Islington who are facing challenges with their social and emotional development. | Schools | Becoming a Man (BAM) is a trauma-informed counselling and group mentoring project integrating clinical theory and practice, men’s rites of passage work, and a dynamic approach to youth engagement. Participation involves weekly group sessions which follow a 30-lesson curriculum delivered in a school setting, group activities outside of school property or school time and support with counsellors. Attendance in at least 13 BAM group sessions improves participants resiliencies, school engagement and relationships. | The SWW delivery and scaling model involves expanding to three additional schools in Lambeth and Islington. |
| Offload | United Kingdom | Men aged 16 and above who are identified to be at risk of mental ill health and are living in Northern England and working in the construction industry. | Workplace | Offload is a mental fitness program for men that was developed by men. It aims to address key mental health areas, including understanding what mental health is and building positive coping strategies. The program is delivered by current and former professional rugby league players as a series of workshops in a male-safe setting (which participants can attend on a drop-in basis) to improve positive coping strategies, meaningful relationships, mental health literacy, help-seeking behaviours and motivation for training/employment. | The delivery of Offload under the SWW program involves expanding the reach to men currently employed in the construction industry in northern England (a high-risk sector and region for mental ill health). Offload engages contractors/workplaces, some large-scale companies and focuses on smaller firms and employers of lower skilled, lower paid workers to participate. |
| Good Vibrations | United Kingdom | Men aged 50 and above in Northern Ireland. | Community | Northern Ireland is reported to have the highest prevalence of poor mental health in the UK, with psychiatric morbidity in Northern Ireland 25% higher than in rest of the UK. The legacy of violence and socio-economic factors are frequently cited as key contributors to poor mental health in the population. Good Vibrations is a men’s health program from aimed specifically at men aged 50 and over. The program aims to improve participants mental health and wellbeing through group-based social participation, online community programs, workshops, guides, and access to free telephone advice. | Delivery of Good Vibrations under the SWW program involves a multi-tiered approach to expand the reach of program services. |
